# L5 spondylolysis increases segmental mobility at the cranial adjacent level without altering intervertebral disc contact pressure

**DOI:** 10.3389/fbioe.2025.1653918

**Published:** 2025-10-02

**Authors:** Zhilin Ge, Bingde Zhao, Xu Xu, Lin Chen, Dongzhu Liang, Qingyang Kang, Zibo Gao, Junhua Luo, Jiheng Zhan, Jianquan Chen, Bo Zhang

**Affiliations:** ^1^ Guangzhou University of Chinese Medicine, Guangzhou, China; ^2^ Guangdong Provincial Hospital of Chinese Medicine, Guangzhou, China; ^3^ Guangdong Provincial Hospital of Chinese Medicine Zhuhai, Zhuhai, China; ^4^ Guangdong Provincial Key Laboratory of Medical Biomechanics, Southern Medical University, Guangzhou, China

**Keywords:** spondylolysis, lumbar, biomechanics, adjacent segment, contact force

## Abstract

**Objective:**

While lumbar spondylolysis has been biomechanically associated with subsequent spondylolisthesis and disc degeneration, its implications on cranial adjacent segments remain unclear. This *in vitro* experiment aims to quantify the segmental alterations in kinematics and contact mechanics at both L5/S1 and L4/L5 levels induced by L5 pars defects.

**Methods:**

Six fresh-frozen human lumbar cadaveric specimens (L1-S2) underwent pure moment loading (4 Nm) in flexion-extension, lateral bending, and axial rotation. Sequential testing compared intact specimens with simulated L5 bilateral spondylolysis models. Intervertebral kinematics were quantified using optical motion tracking, while L4/L5 disc contact parameters were measured using Tekscan pressure sensors.

**Results:**

L5/S1 segmental mobility increased in lateral bending (1.66°, p = 0.002) and axial rotation (1.45°, p = 0.007) in spondylolysis models. Motion increases were observed at the cranial adjacent L4/L5 segment: flexion-extension (1.89°, p < 0.001), lateral bending (2.15°, p = 0.002), and axial rotation (1.89°, p = 0.022). However, no significant differences were detected in the L4/L5 disc contact parameters for peak contact pressure, contact area, and contact force.

**Conclusion:**

Isthmic defects induce segmental hypermobility at the cranial adjacent segment. This kinematic alteration may accelerate disc degeneration.

## Introduction

Spondylolysis of the lumbar vertebrae is considered an anatomical defect of the pars interarticularis caused by both congenital factors and repetitive injuries to the pars (Motley et al., 1998; Fredrickson et al., 1984; Mohile et al., 2022). The condition has a significant prevalence in the population, estimated at approximately 11.5% (Kalichman et al., 2009). Previous studies have reported that 51.4% of patients with spondylolysis also experience spondylolisthesis, compared to only 7.4% in those without spondylolysis, highlighting the strong association between these two conditions (Aoki et al., 2020). The defective segments in spondylolysis, due to shear forces, not only lead to spondylolisthesis but also increase the incidence of disc displacement and degenerative disc disease, which can result in lower back pain and radiculopathy (Mihara et al., 2003; Li et al., 2022). Both of these pathological changes may cause lumbar pain and nerve root symptoms (Niggemann et al., 2011).

To investigate the impact of spondylolysis on the lumbar spine, researchers have explored various aspects such as radiology, biomechanics, and kinematics, yielding numerous reliable conclusions (Takashima et al., 2014; Xu et al., 2024; Ramakrishna et al., 2018; Ichikawa et al., 1982; Melnyk et al., 2013). However, previous research has primarily focused on the caudal segments of spondylolysis, where spondylolisthesis commonly occurs. There is limited research on the adjacent cranial segments of the slipped vertebrae, and there are conflicting results between clinical studies and mechanical experiments (Henson et al., 1987; Zhou et al., 2024). Therefore, to directly measure the biomechanical characteristics of the cranial segments adjacent to spondylolysis, this study conducted *ex vivo* biomechanical experiments to analyze these segments, aiming to provide mechanical insights for predicting disease progression, diagnosis, and treatment in clinical practice.

## Methods

### Sample collection and preparation

Six fresh-frozen lumbar spine cadaveric specimens (provided by the Clinical Anatomy Laboratory of Southern Medical University) with unspecified genders and ages were selected. All specimens underwent visual inspection and CT examination to exclude traumatic injuries, previous infections, tumors, or other signs of bone destruction. Additionally, no significant spinal canal stenosis, osteophyte formation, facet joint hyperplasia, intervertebral space stenosis, disc herniation, or obvious disc degeneration (such as vacuum phenomenon) was observed on the CT images. Obvious deformities such as scoliosis, ankylosing spondylitis, and prior surgical interventions were also rigorously screened. Each lumbar specimen was cataloged individually. After thorough evaluation, soft tissues, including skin, fascia, adipose tissue, and muscles, were carefully removed, preserving the integrity of ligaments, joint capsules, intervertebral discs, and osseous structures. The experimental segments focused on L4/5 and L5/S1. Three to four conventional screws (4.0 mm diameter) were radially inserted into the cranial and caudal vertebral bodies (L1 and S2), with half of the screw length remaining exposed. Specimens were then embedded and fixated within a custom-designed square base using denture base resin. Prepared specimens were wrapped in saline-soaked gauze, sealed in specimen bags, and stored at −20 °C. Before testing, specimens were naturally thawed in a controlled environment with regulated temperature and humidity. To preserve biomechanical integrity, specimens were intermittently moistened with saline spray during preparation and experimental procedures (Chen et al., 2016). All procedures adhered to institutional regulations and ethical guidelines governing cadaveric specimen use.

### Flexibility test

**FIGURE 1:**
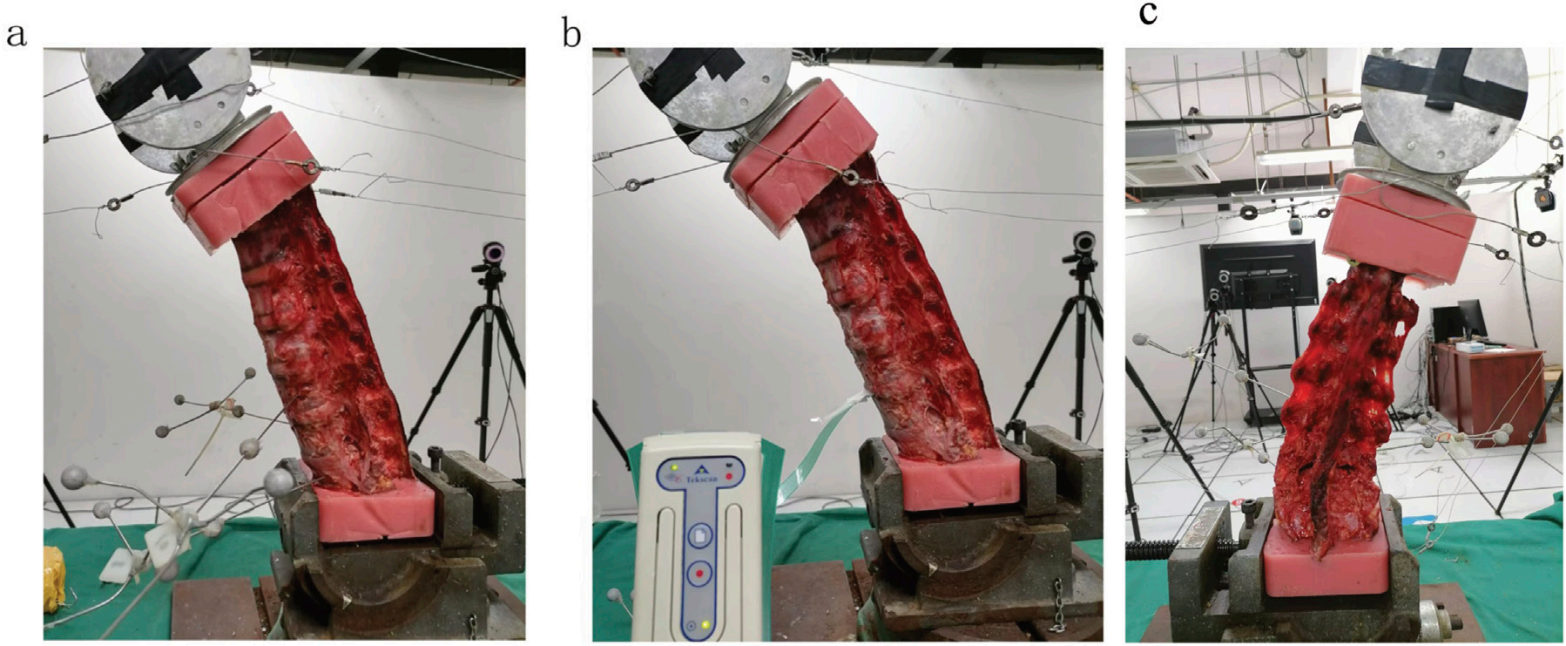
Biomechanical experimental setup: **(a)** Flexibility test: a coupled pulley system applying non-destructive pure moment integrated with an optical motion capture system. **(b)** Contact biomechanics test: digital pressure sensors were placed entirely inside the intervertebral space. **(c)** bilateral L5 pars defect model.

A custom fixture was equipped to secure the marked specimen base to a three-dimensional spinal motion simulator horizontally. The cranial end of the specimen was rigidly attached to a loading platform connected to a pulley system via screws, forming a biomechanical loading system (Kuo and Wu, 2022) (Figure 1a). The system applied a pure moment of 4 N·m to the specimen across six degrees of freedom: flexion-extension, lateral bending, and axial rotation. Three slender rods, each embedded with four marker spheres, were firmly inserted into the anterolateral aspects of the L4–S1 vertebrae. The markers on each rod were aligned within the same plane, resulting in 12 markers to facilitate three-dimensional motion tracking via an optical motion capture system. A motion analysis system (Motion Analysis Corporation) recorded the three-dimensional positional changes of the markers at a sampling rate of 60 Hz, enabling calculation of the range of motion (ROM) under the six loading conditions.

### Contact biomechanics test

A Tekscan digital pressure sensor (K-Scan 6900, Tekscan, Inc., United States) was used to measure intervertebral disc contact biomechanics at the L4/5 level (the cranial adjacent segment to the spondylolytic lesion). The Tekscan 6900 pressure sensor features a 14 mm × 14 mm sensing matrix with a thickness of 0.102 mm, a spatial resolution of 62.0 sensels/cm^2^, and a maximum pressure capacity of 68,948 kPa. Based on established protocols from previous studies in our laboratory, sensor calibration was conducted as follows: A BOSE ElectroForce dynamic/static materials testing system (Model 3510-AT, United States; System ID: 100273) was employed. The sensor was interfaced with a Tekscan connection hub and integrated with a computer running I-Scan software to verify signal acquisition functionality. Following horizontal positioning on the loading platform, initial contact was adjusted to achieve zero-load baseline conditions. Stepwise vertical loads (0 N, 50 N, 100 N, and 150 N) were applied during calibration while synchronously recording machine-derived force-time data and I-Scan output signals. This procedure enabled the derivation of a calibration curve correlating sensor outputs with actual force measurements, with resultant calibration files archived (Ma et al., 2025). For subsequent biomechanical testing, dedicated sensors were assigned to individual specimens to mitigate potential sensitivity attenuation, thereby ensuring metrological accuracy and validity. The sensor was preconditioned in normal saline for 48 h before testing to minimize linear output drift during measurements (Park et al., 2023). A transverse incision approximately 3 cm in width and 2.5 cm in depth was created along the anterior midline of the L4/5 disc to ensure complete sensor accommodation within the intervertebral compartment. The sensor was carefully positioned to prevent deformation while maintaining close contact with the prepared surfaces of the incision. An assistant manually stabilized the sensor’s trailing cable to prevent dislodgment during testing (Figure 1b). Each specimen underwent calibration according to the manufacturer’s guidelines before parameters were recorded via pressure-mapping software during six degrees of freedom moment loading.

### Test protocol

**FIGURE 2:**
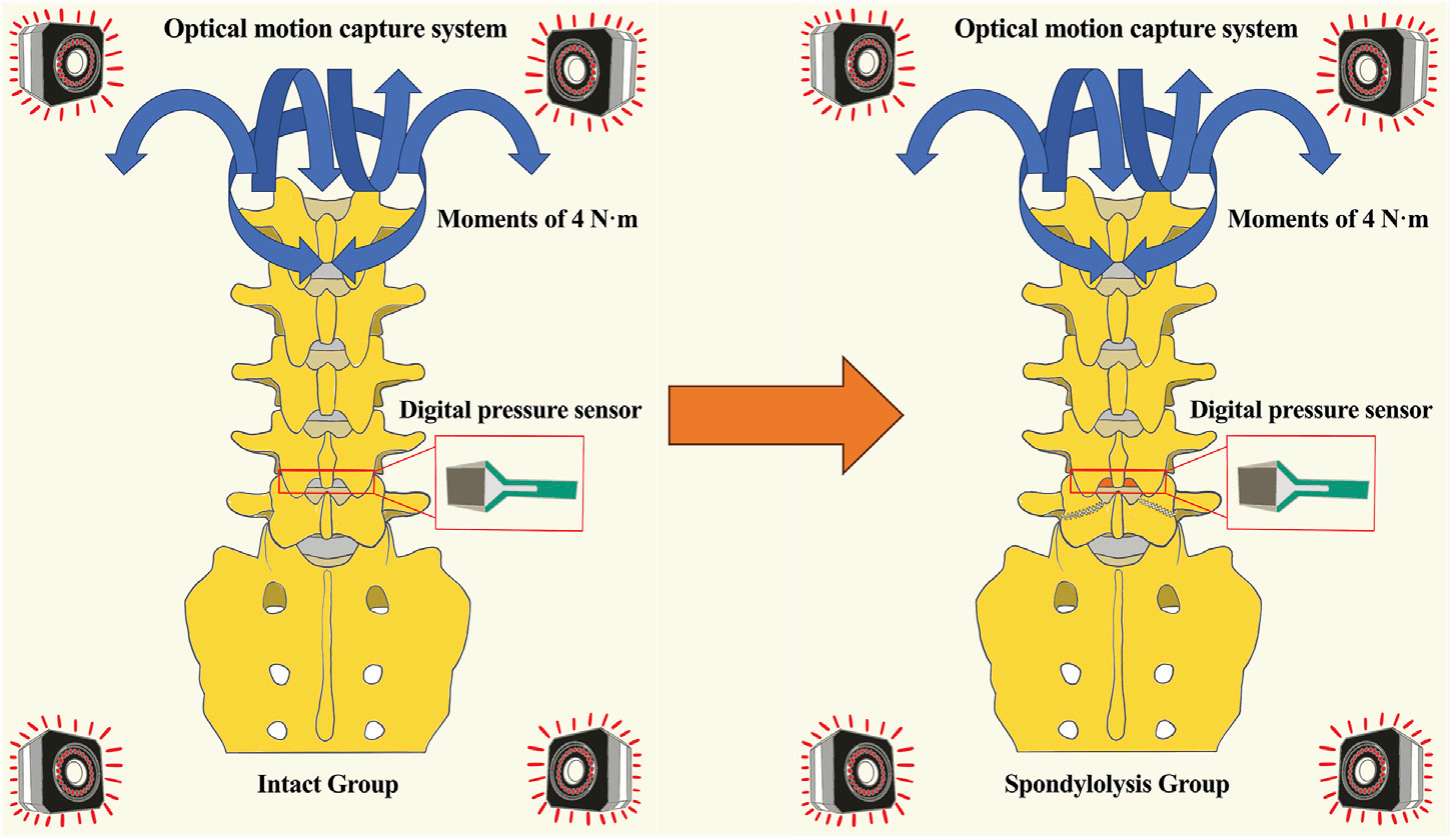
Diagram comparing “Intact Group” and “Spondylolysis Group” spine models. Both models display pressure sensors and an optical motion capture system, with blue arrows indicating moments of four newton-meters. An orange arrow indicates the surgical creation of a bilateral pars defect in the tested specimen, converting it from the Intact to the Spondylolysis condition.

The intact condition preserved native anatomical structures, including intervertebral discs, anterior/posterior longitudinal ligaments, ligamentum flavum, interspinous/supraspinous ligaments, facet joint capsules, and articular cartilage. Spondylolysis simulation was initiated following ROMs and disc pressure measurements in the intact state. Bilateral L5 interarticular pars defects were surgically created using a bone saw. The location where we osteotomized the isthmus of the vertebral arch using a bone saw was: from the midpoint of the cranial lamina of L5 between the L4/5 facet joint and the spinous process, extending laterally and caudally through the inferomedial margin of the pedicle (to avoid pedicle injury), reaching the lateral margin of the L5 inferior articular process. Throughout the procedure, ligaments and facet joints were meticulously protected (Figures 1c, 2). Three loading cycles were applied in each direction to minimize viscoelastic effects, with the first two cycles serving as preconditioning and the third cycle used for kinematic data acquisition. All spinal specimens underwent sequential biomechanical testing under two conditions: (1) intact state (intact group) and (2) simulated bilateral L5 spondylolysis (spondylolysis group) (Figure 2).

### Statistical analysis

Statistical analyses were conducted using SPSS v29.0 for Mac (IBM Corp, Armonk,NY). Continuous variables were presented as mean ± standard deviation (SD). Paired Student’s t-test was performed for comparisons between the intact group and spondylolysis group. Statistical significance was defined as a two-tailed P-value <0.05.

## Results

**FIGURE 3:**
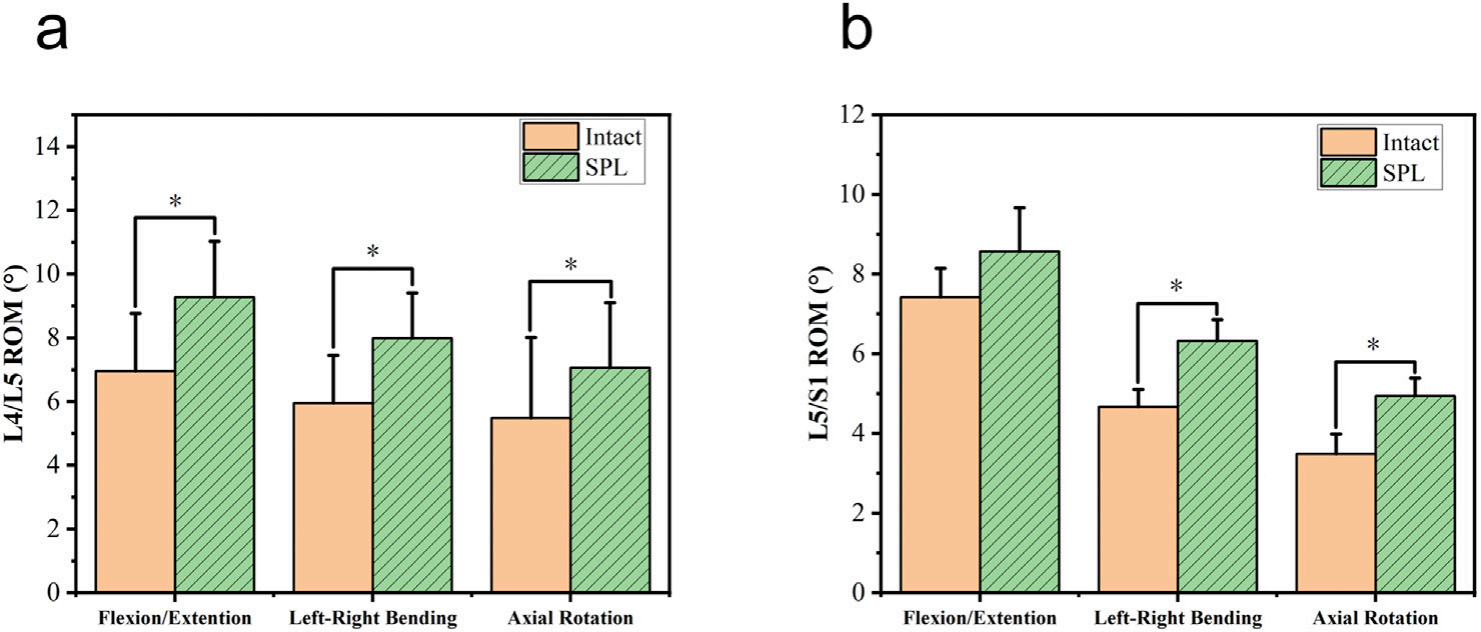
Comparison of range of motion (ROM) in degrees for L4/L5 and L5/S1 segments. Graph a shows higher ROM in SPL (green) compared to intact (orange) for flexion/extension, left-right bending, and axial rotation, with significant differences. Graph b shows similar patterns for L5/S1. SPL: spondylolysis. *: Compared with the intact group, *P*<0.05.

**FIGURE 4:**
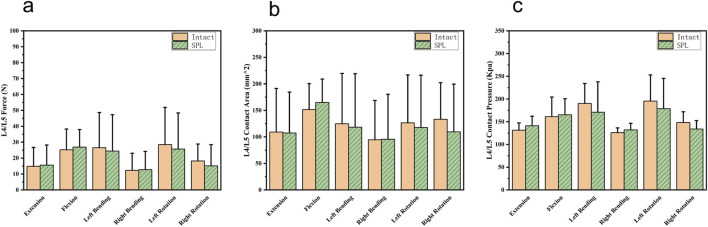
Comparison of cranial adjacent segments (L4/L5) contact biomechanical parameters between the intact and spondylolysis groups under the six loading conditions. **(a)** Similar contact forces were observed in all six loading conditions. **(b)** Contact areas were comparable. **(c)** Contact pressures exhibited nearly identical values. SPL: spondylolysis.

One specimen failed to yield data during the contact biomechanics test due to apparatus damage. Since the missing data exhibited completely random missingness, and this experiment employed a self-controlled paired test design, our approach was to directly remove all contact parameters from this specimen, excluding them entirely from statistical analysis without imputation. This *in vitro* biomechanical study investigated whether L5/S1 spondylolysis alters motion patterns and mechanical loading at the cranial adjacent segment (L4/L5). The kinematic data, including the differences in ROM between the intact model (baseline pre-experimental values) and the isthmic spondylolysis model, are summarized in Table 1. Motion analysis demonstrated that L5/S1 spondylolysis significantly increased the range of motion (ROM) at the L5/S1 level, with increments in lateral bending (1.66°, p = 0.002; Figure 3a) and axial rotation (1.45°, p = 0.007; Figure 3a). At the L4/L5 level, spondylolysis at L5/S1 induced substantial ROM increases across multiple planes: flexion-extension (1.89°, p < 0.001; Figure 3b), lateral bending (2.15°, p = 0.002; Figure 3b), and axial rotation (1.89°, p = 0.022; Figure3b). However, compressive load analysis revealed no significant differences in intervertebral contact force at the L4/L5 level between the two groups (p > 0.05; Figure 4a). Furthermore, neither contact area (Figure 4b) nor contact pressure (Figure 4c) at the L4/L5 intervertebral space exhibited statistically significant alterations attributable to L5/S1 spondylolysis. The contact biomechanical outcomes are summarized in Table 2.

**TABLE 1:** Comparison of kinematic parameters between the intact group and the spondylolysis group.

	Intact group	Spondylolysis group	ΔROM
Flexion extension			
L4/5 (°)	6.95 ± 1.81	9.27 ± 1.76*	1.89 ± 0.73
L5/S1 (°)	7.42 ± 0.73	8.57 ± 1.09	1.15 ± 1.14
Lateral bending			
L4/5 (°)	5.95 ± 1.50	7.99 ± 1.42*	2.15 ± 0.79
L5/S1 (°)	4.67 ± 0.44	6.33 ± 0.53*	1.66 ± 0.69
Axial rotation			
L4/5 (°)	5.49 ± 2.53	7.06 ± 2.04*	1.89 ± 1.05
L5/S1 (°)	3.49 ± 0.49	4.94 ± 0.45*	1.45 ± 0.79

*Compared with Intact Group, P < 0.05. ROM: range of motion.

**TABLE 2:** Comparison of contact biomechanical outcomes between the intact group and the spondylolysis group.

Parameters	Intact group	Spondylolysis group
Extension		
Force (N)	14.86 ± 11.87	15.59 ± 12.60
Contact area (mm^2)	109.20 ± 82.11	107.40 ± 77.14
Contact pressure (Kpa)	131.35 ± 16.04	141.05 ± 21.26
Flexion		
Force (N)	25.19 ± 13.11	26.94 ± 11.02
Contact area (mm^2)	151.60 ± 48.87	165.00 ± 44.16
Contact pressure (Kpa)	161.18 ± 43.20	165.35 ± 35.30
Left bending		
Force (N)	26.54 ± 22.14	24.48 ± 22.80
Contact area (mm^2)	124.80 ± 94.89	118.40 ± 100.84
Contact pressure (Kpa)	190.13 ± 44.15	171.02 ± 66.76
Right bending		
Force (N)	12.28 ± 10.78	12.77 ± 11.45
Contact area (mm^2)	94.60 ± 47.11	95.60 ± 84.75
Contact pressure (Kpa)	126.03 ± 10.51	132.02 ± 14.58
Left rotation		
Force (N)	28.52 ± 23.28	25.69 ± 22.68
Contact area (mm^2)	126.60 ± 90.29	117.60 ± 98.85
Contact pressure (Kpa)	195.47 ± 57.66	178.64 ± 66.65
Right rotation		
Force (N)	18.19 ± 10.68	15.11 ± 13.36
Contact area (mm^2)	133.40 ± 68.85	109.60 ± 89.85
Contact pressure (Kpa)	148.29 ± 23.74	134.02 ± 18.48

## Discussion

Lumbar spondylolysis is an important factor causing lumbar spondylolisthesis and intervertebral disc degeneration (Sima et al., 2024). Previous studies primarily focused on the caudal segments of spondylolysis, while investigations of the cranial segments mainly addressed adjacent segment disease following spinal fusion surgery (Nakajima et al., 2024; Okuda et al., 2018). However, Jeong et al. (Jeong et al., 2013) noticed that high-intensity zones (HIZ) in intervertebral discs were more frequently identified at cranial segments of spondylolysis, suggesting potential influences on adjacent disc degeneration. Henson et al. (Henson et al., 1987) reported that 44% of L5/S1 spondylolytic spondylolisthesis patients exhibited L4/5 retrolisthesis, with 51% demonstrating L4/5 disc disruption during discography, half of whom reproduced symptomatic pain upon injection. A recent clinical study by Zhou et al. (Zhou et al., 2024) revealed that 14.9% of grade I/II L5/S1 spondylolytic spondylolisthesis cases concurrently presented L4/5 disc herniation, emphasizing the necessity to consider cranial adjacent discs in diagnosis and treatment. These clinical outcomes collectively indicate that lumbar spondylolysis may have potential influences on the degeneration of cranial adjacent discs. Therefore, for patients with early-stage spondylolysis or candidates for conservative treatment, degeneration trends in both defective and adjacent segments should be evaluated to formulate preventive strategies, therapeutic approaches, and follow-up protocols. This study supplements the current understanding of biomechanical alterations in cranial adjacent intervertebral spaces.

In this study, the spondylolysis group exhibited increased ROMs at both defective and adjacent segments compared to intact models. This aligns with findings by Phan et al. (Phan et al., 2015) using dynamic MRI, which identified adjacent segment instability in 49% of spondylolisthesis cases at L4/5, 34% at L5/S1, and 23% at L3/4. Mehta et al. (Mehta et al., 2012) clinically observed L4 retrolisthesis in 29% of L5 spondylolytic spondylolisthesis patients. Progressive degeneration scores from L3/4 through L4/5 to L5/S1 suggest that L5 spondylolytic spondylolisthesis significantly contributes to degeneration and abnormal motion in the cranial adjacent segment. Previous studies have implicated abnormal ROM increases as potential kinematic factors for disc degeneration, possibly through enhanced mechanical loading (Prasarn et al., 2012; Eck et al., 2002). Thus, increased ROM at cranial segments may accelerate disc degeneration via excessive loading. Biomechanical studies have yielded conflicting evidence regarding spinal motion characteristics in this condition. In a radiographic investigation utilizing early biplanar X-ray imaging systems, Pearcy et al. (Pearcy and Shepherd, 1985) demonstrated diminished segmental lumbar ROMs in patients with bilateral pars defects presenting spondylolytic spondylolisthesis. This reduction was hypothesized to result from compromised biomechanical efficiency in degenerated intervertebral discs. Furthermore, the degenerated discs may induce altered kinematic patterns in adjacent spinal segments, potentially accelerating adjacent segment degeneration through abnormal motion transmission. Consequently, this degenerative cascade could lead to progressive ROM reduction in adjacent spinal segments over time (Cai et al., 2020; Wang et al., 2023; Zirbel et al., 2013). These findings collectively suggest that spondylolysis not only induces segmental instability and disc degeneration but may also accelerate adjacent segment degeneration.

In our work, biomechanical analysis revealed no significant elevation in adjacent segment intervertebral pressure across spondylolytic defect models. Finite element analyses examining different fixation procedures similarly reported no substantial elevation in the maximum von Mises stress at cranial adjacent discs of spondylolysis models compared to intact models (Ye et al., 2022; Pan et al., 2023), consistent with our findings. However, since the models used in this study and those in published finite element analysis studies by other teams derive from distinct specimens, and discrepancies exist in loading protocols, direct comparison between the outcomes may be constrained. Given the absence of prior mechanical experiments investigating analogous mechanisms, this cross-comparison serves as a reference. The observed ROM increase at L4/5 in spondylolysis models might reflect altered loading patterns. Animal studies have established shear stress as a critical contributor to disc degeneration (Kim et al., 2012; Xia et al., 2015). Compared to conventional needle-type pressure sensors, the thin-film electronic pressure sensors employed in this study enable more precise acquisition of contact area, force, and pressure distribution, making more comprehensive biomechanical datasets available. It should be noted, however, that both sensor modalities primarily measure axial stress within intervertebral discs (Park et al., 2023; Gay et al., 2008). Considering our findings of increased adjacent segment ROM without axial stress elevation, we hypothesize that spondylolysis may primarily increase shear stress in cranial adjacent discs.

Our findings provide quantifiable evidence that spondylolysis-induced motion redistribution is a critical etiological factor in adjacent segment disease pathogenesis, offering clinicians a suggestion for evaluating degenerative risks in pars defect patients. During the management of patients with isthmic spondylolysis, clinicians should also consider that the cranial adjacent segment may experience accelerated degeneration secondary to the defect, potentially evolving into a symptomatic segment. Close monitoring through regular follow-up evaluations is therefore essential, with tailored radiological or imaging strategies implemented to assess the progression of segmental degeneration.

### Limitations

Some limitations to this study should be addressed. Legal restrictions on cadaveric experiments limited specimen acquisition to natural death donors, potentially introducing variability in disc degeneration status. However, due to institutional restrictions, age data was inaccessible to our team, and MRI data for Pfirrmann classification could not be obtained as we were a collaborating institution without authorization for additional imaging. Furthermore, both needle-type and thin-film pressure sensors require partial disc disruption during implantation, potentially altering mechanical efficiency. Advanced non-invasive measurement techniques are needed to address these challenges in intervertebral pressure assessment. Furthermore, due to apparatus damage during testing, all contact biomechanical parameters from one specific specimen were excluded from statistical analysis. This reduction in sample size inevitably diminishes statistical power. This is documented with the expectation that future studies may include more specimens or advanced measurement techniques to strengthen our conclusions.

## Conclusion

Through a multimodal approach combining optical motion capture and pressure sensor analysis in lumbar cadaveric specimens, this study demonstrates that isthmic defects induce segmental hypermobility at the cranial adjacent segment. This kinematic alteration may initiate a biomechanical cascade culminating in disc degeneration.

## Data Availability

The raw data supporting the conclusions of this article will be made available by the authors, without undue reservation.
